# Role of Corticotropin-Releasing Factor (CRF) Receptor-1 on the Catecholaminergic Response to Morphine Withdrawal in the Nucleus Accumbens (NAc)

**DOI:** 10.1371/journal.pone.0047089

**Published:** 2012-10-10

**Authors:** Pilar Almela, Javier Navarro-Zaragoza, Juan-Antonio García-Carmona, Lucía Mora, Juana Hidalgo, María-Victoria Milanés, María-Luisa Laorden

**Affiliations:** Departamento de Farmacología, Facultad de Medicina, Universidad de Murcia, Murcia, Spain; Karolinska Inst, Sweden

## Abstract

Stress induces the release of the peptide corticotropin-releasing factor (CRF) into the ventral tegmental area (VTA), and also increases dopamine (DA) levels in brain regions receiving dense VTA input. Since the role of stress in drug addiction is well established, the present study examined the possible involvement of CRF1 receptor in the interaction between morphine withdrawal and catecholaminergic pathways in the reward system. The effects of naloxone-precipitated morphine withdrawal on signs of withdrawal, hypothalamo-pituitary-adrenocortical (HPA) axis activity, dopamine (DA) and noradrenaline (NA) turnover in the nucleus accumbens (NAc) and activation of VTA dopaminergic neurons, were investigated in rats pretreated with vehicle or CP-154,526 (selective CRF1R antagonist). CP-154,526 attenuated the increases in body weight loss and suppressed some of withdrawal signs. Pretreatment with CRF1 receptor antagonist resulted in no significant modification of the increased NA turnover at NAc or plasma corticosterone levels that were seen during morphine withdrawal. However, blockade of CRF1 receptor significantly reduced morphine withdrawal-induced increases in plasma adrenocorticotropin (ACTH) levels, DA turnover and TH phosphorylation at Ser40 in the NAc. In addition, CP-154,526 reduced the number of TH containing neurons expressing c-Fos in the VTA after naloxone-precipitated morphine withdrawal. Altogether, these results support the idea that VTA dopaminergic neurons are activated in response to naloxone-precipitated morphine withdrawal and suggest that CRF1 receptors are involved in the activation of dopaminergic pathways which project to NAc.

## Introduction

Addiction research has traditionally focused on dopamine (DA) and positive reinforcement-based behaviours. However, increased focus has been placed on negative reinforcement as a key driver in the addiction process. Noradrenergic and corticotropin-releasing factor (CRF) signalling systems have been heavily implicated in negative reinforcement [1]–[3]. Both noradrenaline (NA) and CRF are critical in behavioural aspects of addiction, including the reinforcing properties of drugs [4], [5] and anxiogenic effects of drug withdrawal [6], [7]. CRF is an important regulator of stress response that exerts its actions through activation of two different types of G-protein-coupled receptors: CRF1 (expressed throughout the entire central nervous system) and CRF2 (displays more restrictive expression that CRF1) [8]. CRF1 binding sites have been demonstrated in several key brain areas involved in the addictive processes [e.g., cerebral cortex, hippocampus, hypothalamus, amygdala, nucleus of tractus solitarius (NTS), ventral tegmental area (VTA) and nucleus accumbens (NAc) that are involved in reward, reinforcement, craving and aversive effects of drugs of abuse [9]. Moreover, the decreased brain reward function associated with drug withdrawal is CRF1 receptor-dependent [10].

Enhanced responsiveness of hypothalamo-pituitary-adrenocortical (HPA) axis after morphine withdrawal, which results in an increase in CRF transcription and boost of adrenocorticotropin and corticosterone secretion, has been associated with activation of noradrenergic neurons in the NTS that project to the hypothalamic paraventricular nucleus (PVN) [11], [12]. CRF is also located outside the HPA axis to control autonomic and behavioural responses to stressors. NA would modulate the release of CRF in the brain stress system, including the central amygdala, the bed nucleus of stria terminalis and the PVN of the hypothalamus. CRF from these nuclei would induce the release of NA by the brain stem noradrenergic areas [13], [14]. In addition, the NAc and its dopaminergic inputs from the VTA is one of the most important anatomical substrates for drug reward and aversion [15], [16]. Mu-opioid receptor agonists increase DA release in terminal regions in the NAc by inhibiting GABAergic neurons in the VTA, which provide tonic inhibition of DA neurons [17]. Research indicates that midbrain DA neurons not only show a pattern signaling the magnitude, delay and probability of rewards [18], [19] but also code negative motivation and aversive events [20]. Stress can induce relapse in addicted or abstinent humans [21] and reinstate drug seeking in animal models of relapse [22]. Since stress not only increases DA release in brain regions receiving dense VTA input [23]–[26] but also stimulates the release of CRF into the VTA [27], it has been suggested that CRF may directly excite the midbrain DA system [28].

Altogether, these results suggest the existence of a DA/NA-CRF loop; however, the possible involvement of CRF receptor subtypes in the interaction between morphine withdrawal and catecholaminergic pathways in the reward system is not well documented. Therefore, here we examined: 1) the role of CRF1 receptor in mediating somatic and behavioural states produced during withdrawal from morphine dependence, 2) the activation of HPA axis induced by morphine withdrawal in morphine dependent rats pretreated with a CP-154,526, a selective CRF1 receptor antagonists, 3) the response of dopaminergic and noradrenergic pathways innervating the NAc and the effects of CRF1 receptor blockade on tyrosine hydroxylase (TH) phosphorylation in Serine (Ser)40 and Ser31 in the NAc and 4) the effects of CRF1 receptor blockade on activation of VTA dopaminergic neurons during morphine withdrawal, as reflected by c-Fos expression.

## Results

**Figure 1:**
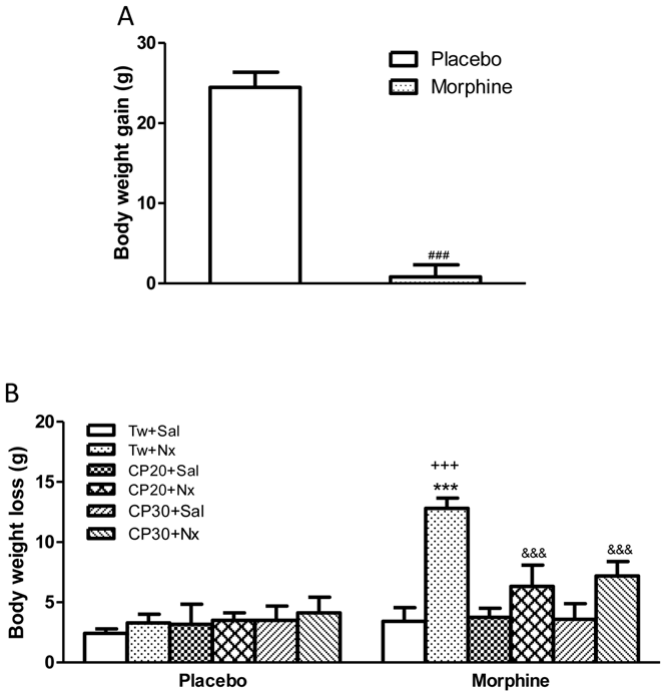
(A) Body weight gain after s.c. implantation of placebo or morphine (75 mg) pellets. (B) Rats were injected with Tween 80 (Tw) or CP-154,526 (20 or 30 mg/kg, i.p., CP) 30 min before saline (Sal) or naloxone (1 mg/kg, s.c., Nx) to evaluate body weight loss. Data are the mean±SEM (n = 5–28). ###p<0.001 versus placebo; ***p<0.001 versus morphine+Tw+Sal; +++ p<0.001 versus placebo+Tw+Nx; &&&p<0.001 versus morphine+Tw+Nx.

In accordance with previous findings, Student's t-test showed that rats receiving long-term morphine treatment had significantly lower body weight gain (Fig. 1A) which might be due to the reduced food intake observed during morphine treatment [29].

### Effects of CRF1 receptor antagonist on naloxone-precipitated withdrawal signs in morphine dependent rats

Two-way ANOVA showed that there was a significant morphine pretreatment main effects [F(1,63) = 20.49, p<0.0001], a significant naloxone main effect [F(5,63) = 6.09, p = 0.0001] and a significant interaction between chronic pretreatment x acute treatment [F(5,63) = 5.09, p = 0.0006]. Newman Keuls' post hoc test showed that naloxone injection to morphine dependent rats pretreated with vehicle produced a significant (p<0.001) increase in body weight loss compared with the control pellet-treated group also receiving naloxone and with morphine treated rats given saline (Fig. 1B). However, administration of naloxone to control rats resulted in no significant changes in body weight loss 1 h after drug injection compared with control rats receiving saline. A significant decrease (p<0.001) in body weight loss was seen in morphine withdrawn rats pretreated with the CRF1 receptor antagonist CP-154,526 (20, 30 mg/kg i.p.) compared with morphine withdrawn rats receiving vehicle instead of CP-154,526.

Naloxone-precipitated withdrawal also caused characteristic signs of abnormal behaviour, such as ptosis, teeth-chattering, tremor, piloerection, lacrimation, rinorrhea, spontaneous jumping, wet-dog shakes, salivation and diarrhoea. A significant lower frequency or total suppression of four of the ten signs (rinorrhea, spontaneous jumping, salivation and diarrhoea) was noted in morphine dependent rats pretreated with CP-154,526 (20 or 30 mg/kg i.p.) before naloxone (Table 1).

**Table 1:** Behavioural profiles after morphine withdrawal precipitated by naloxone (nx) in animals chronically administered with vehicle (tween80) or CP-154,526 (CP).

Withdrawal signs	Vehicle+nx	CP20+nx CP30+nx
**Ptosis**	2/7	0/5 0/5
**Teeth-chatering**	7/7	2/5 3/5
**Tremor**	3/7	2/5 2/5
**Piloerection**	0/7	0/5 0/5
**Lacrimation**	2/7	0/5 1/5
**Rinorrhea**	6/7	0/5* 0/5*
**Spontaneous jumping**	6/7	0/5* 0/5*
**Wet-dog shakes**	4/7	3/5 3/5
**Salivation**	7/7	0/5** 0/5**
**Diarrhoea**	5/7	0/5* 0/5*

* *p*<0.05,

** *p*<0.01 versus vehicle+nx (X^2^ test). Animals received subcutaneous implantation of morphine (75 mg) pellets. On day 6 rats were injected with vehicle or CP-154,526 (20 or 30 mg/kg, i.p.) 30 min before naloxone (1 mg/kg, s.c.) and were decapitated 60 min after the opioid antagonist administration. The behaviours are shown as the number of animals exhibiting the withdrawal signs of the total number of animals observed for 60 min.

### Effects of CRF1 receptor blockade on morphine withdrawal-induced HPA axis activation

**Figure 2:**
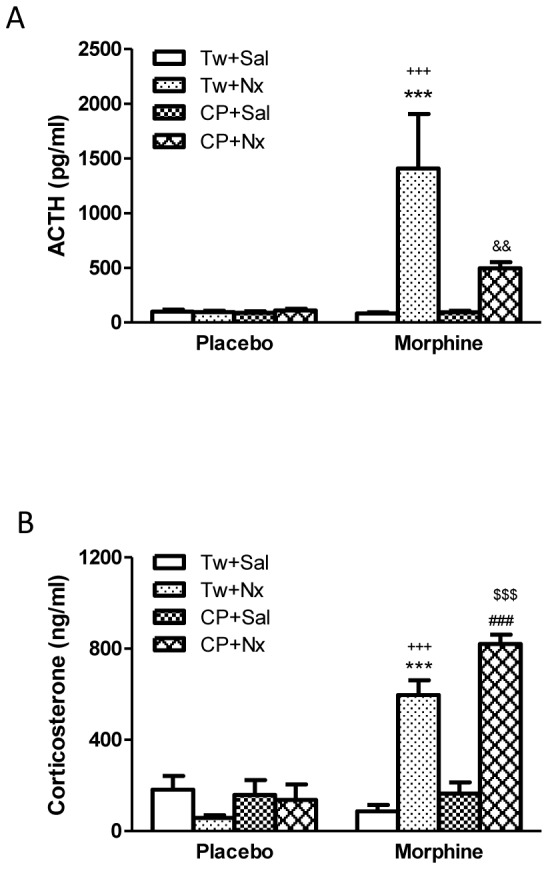
Plasma adrenocorticotropic hormone (ACTH) (A) and corticosterone (B) concentrations 60 min after saline (Sal) or naloxone (Nx) administration to placebo or morphine dependent rats pretreated with Tween 80 (Tw) or CP-154,526 (20 mg/kg, i.p., CP). Data are the mean±SEM (n = 6–9). ***p<0.001 versus the morphine dependent group receiving saline instead of naloxone; +++p<0.001 versus the placebo group injected with Tw+Nx; &&p<0.01 versus morphine+Tw+Nx; ###p<0.001 versus morphine+CP+Sal; $$$p<0.001 versus placebo+CP+Nx.

We measured plasma ACTH and corticosterone concentrations (as HPA axis activation markers) in blood samples obtained from morphine dependent or control rats 60 min after injection of saline or naloxone. Two-way ANOVA indicated that there was a significant morphine pretreatment main effects [ACTH: F(1,52) = 9.59, p = 0.0031; corticosterone: F(1,47) = 52.56, p<0.0001)], significant acute treatment [ACTH: F(3,52) = 5.71, p = 0.0019; corticosterone: F(3,47) = 16.53 p<0.0001)], a significant interaction between chronic pretreatment and acute treatment, [ACTH: F(3,52) = 5.69, p = 0.0019; corticosterone: F(3,47) = 23.88, p<0.0001)]. As shown in Fig. 2A and B, naloxone-precipitated morphine withdrawal evoked a dramatic increase of both ACTH and corticosterone secretion (p<0.001). To evaluate whether a causal link exists between CRF1 receptor activation and HPA axis hyperactivation during morphine withdrawal, we measured plasma ACTH and corticosterone concentrations in animals made dependent on morphine and pretreated with CP-154,526 (20 mg/kg, i.p.) 30 min before naloxone administration. CP-154,526 significantly (p<0.001) reduced morphine withdrawal-induced increases in plasma ACTH compared with rats receiving vehicle instead of CP-154,526, whereas plasma ACTH levels in morphine-treated rats given saline or placebo-treated rats receiving saline or naloxone were not modified by CP-154,526. However, naloxone-precipitated morphine withdrawal produced a significant (p<0.001) increase in corticosterone levels in animals receiving CP-154,526 compared with morphine-treated rats receiving CP-154,526+saline and placebo rats treated with the CRF1 receptor antagonist+naloxone.

### Effects of CRF1 receptor antagonist on naloxone-induced DA and NA turnover and TH phosphorylation in the NAc

**Figure 3:**
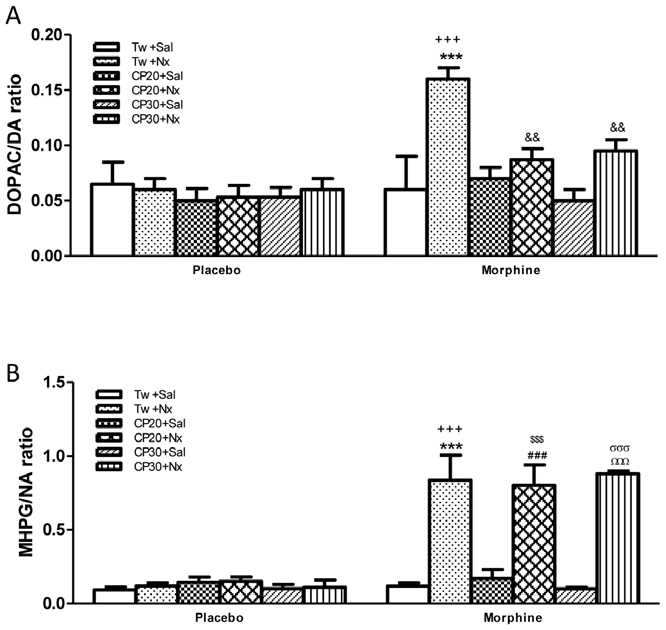
DOPAC/DA (A) and MHPG/NA (B) ratio in the nucleus accumbens (NAc) 60 min after saline (Sal) or naloxone (Nx) administration to placebo or morphine dependent rats pretreated with Tween 80 (Tw) or CP-154,526 (20 or 30 mg/kg, i.p., CP) (A, B, C,). Data are the mean±SEM (n = 5). ***p<0.001, versus the morphine dependent group receiving saline instead of naloxone; +++*p*<0.001 versus the placebo group injected with Tw+Nx; &&p<0.01 versus morphine+Tw+Nx; ###p<0.001 versus morphine+CP20+Sal; $$$p<0.001 versus placebo+CP20+Nx; ΩΩΩp<0.001 versus morphine+CP30+Sal; σσσp<0.001 versus placebo+CP30+Nx.

Analysis of DOPAC/DA and NMN/NA ratio revealed a chronic drug effect [DOPAC/DA: F(1,48) = 14.10, p = 0.0005; MHPG/NA: F(1,48) = 80.44, p<0.0001], an opioid withdrawal effect [DOPAC/DA: F(5,48) = 4.41, p = 0.0022; MHPG/NA: F(5,48) = 16.07, p<0.0001] and a chronic drug treatment x opioid withdrawal [DOPAC/DA: F(5,48) = 3.80, p = 0.0056; MHPG/NA: F(5,48) = 14.80, p<0.0001]. Post hoc test revealed that 60 min after naloxone administration to morphine dependent rats there was an increase (p<0.001) in the DOPAC/DA and MHPG/NA ratio (as index of NA turnover) when compared with morphine dependent rats receiving saline instead of naloxone or naïve rats injected with naloxone (Fig. 3A,B). However, administration of naloxone to placebo-pelleted rats resulted in no significant changes in DA and NA turnover 60 min after drug injection compared with control rats receiving saline. As shown in Fig. 3A, the administration of CP-154,526 (20 or 30 mg/kg) significantly (p<0.01) decreased DOPAC/DA ratio versus morphine withdrawn rats receiving vehicle instead of CP-154,526. No alterations in NA turnover in the NAc were found after naloxone-precipitated morphine withdrawal in rats pretreated with CP-154,526 (Fig. 3B).

**Figure 4:**
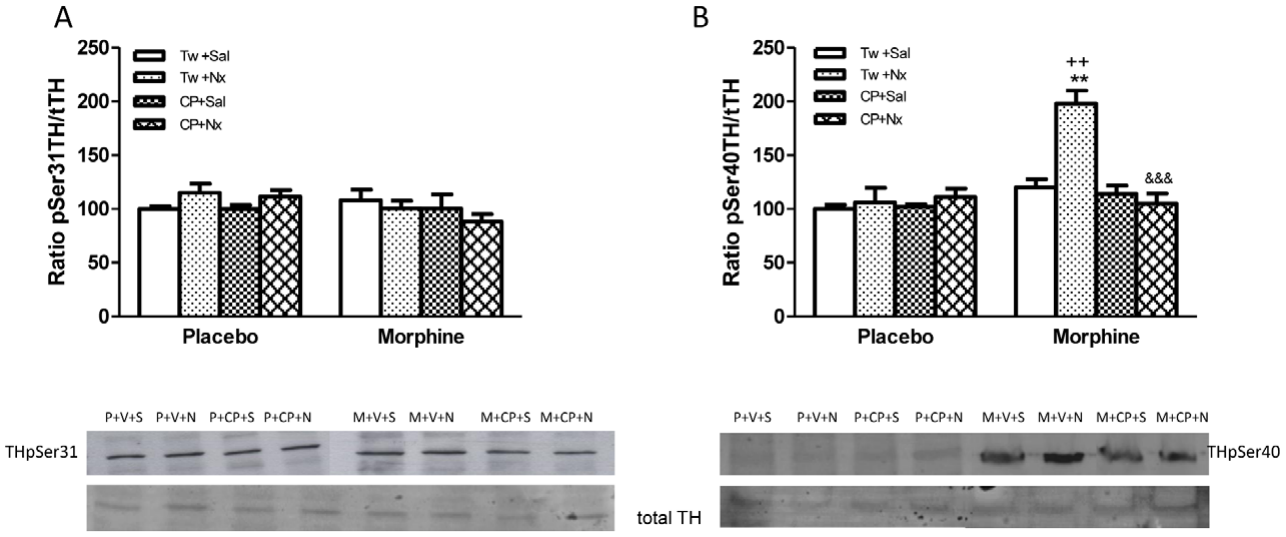
Western-blotting analysis of TH phosphorylated (p) at Ser 31 (A) or Ser40 (B)/total (t)TH ratio in the nucleus accumbens (NAc) 60 min after saline (Sal, S) or naloxone (Nx, N) administration to placebo (P) or morphine (M) dependent rats pretreated with Tween 80 (Tw, V) or CP-154,526 (20 mg/kg, i.p., CP) (A, B). The immunoreactivity corresponding to TH phospho-Ser31 or TH phospho-Ser40 is expressed as a percentage of that in the control group defined as 100% value. Data are the mean±SEM (n = 6). **p<0.01, versus the morphine dependent group receiving saline instead of naloxone; ++p<0.01 versus the placebo group injected with Tw+Nx; &&&p<0.001 versus morphine+Tw+Nx.

In the present work we also examined TH phosphorylation at Ser31 and Ser40. Two-way ANOVA performed on TH phosphorylation at Ser31 indicated that there was a not significant morphine pretreatment main effect, no significant naloxone main effect nor a significant interaction. However, two-way ANOVA results for TH phosphorylated at Ser40 revealed a significant effects of morphine chronic pretreatment [F(1,40) = 22.22, p<0.0001], acute treatment [F(3,40) = 12, p<0.0001] and a significant interaction between chronic pretreatment and acute treatment [F(3,40) = 11.80, p<0.0001]. As shown in Fig. 4A, TH phosphorylated at Ser31 was unchanged in morphine dependent rats treated with vehicle+naloxone or CP-154,526+naloxone. However, we observed an significant (p<0.01) enhancement in the immunoreactivity corresponding to TH phosphorylated at Ser40 in the NAc from morphine dependent rats injected with naloxone (Fig. 4B) versus morphine dependent rats receiving saline instead of naloxone or placebo groups injected with naloxone. Pretreatment with CP-154,526 (20 mg/kg i.p.) significantly (p<0.001) modified the increase in the number of TH cells phosphorylated at Ser40 after naloxone-precipitated morphine withdrawal that was seen in NAc (Fig. 4B).

### Induction of c-Fos in total TH positive neurons in the VTA

**Figure 5:**
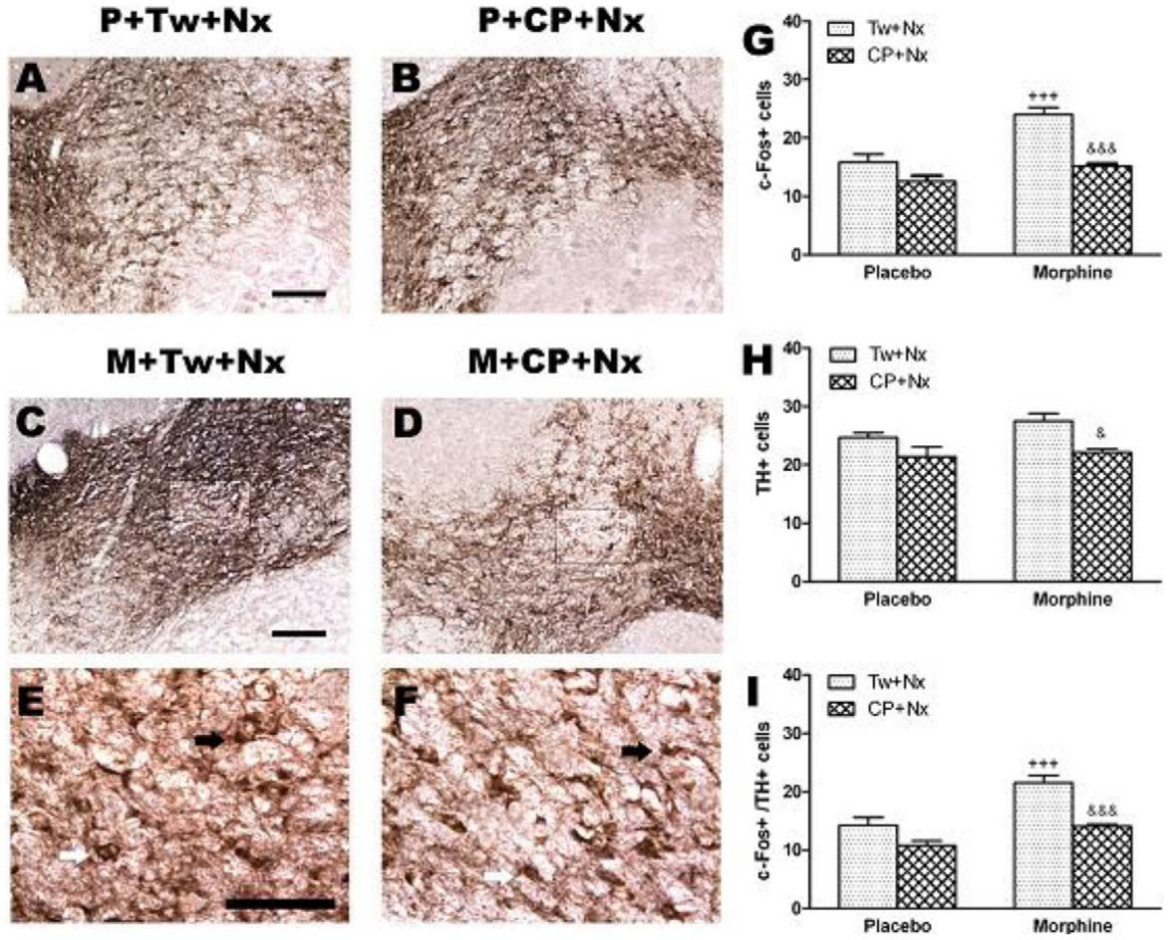
Photographs (A,B,C,D,E,F) represent TH positive neurons coexpressing c-Fos in the ventral tegmental area (VTA) 60 min after naloxone (Nx, N) administration to placebo (P) or morphine (M) dependent rats pretreated with Tween 80 (Tw) or CP-154,526 (20 mg/kg, i.p., CP). Black arrows: c-Fos+/TH+ neurons; white arrows: c-Fos-/TH+ neurons (E,F). Quantitative analysis of the number c-Fos+, TH+ or c-Fos +/TH+ neurons (G,H,I). Data are expressed as the mean ± SEM (n = 6–7). +++*p*<0.001 versus the placebo group injected with Tw+Nx; &p<0.05, &&&*p*<0.001 versus morphine+Tw+Nx.

c-Fos protein, the product of c-fos immediate early gene, has been used as a marker for neuronal activation (Fig. 5 A, B, G). Two-way ANOVA for c-Fos expression revealed a main effect of morphine pretreatment [F(1,15) = 27.05, p = 0.0001] acute treatment [F(1,15) =  34.23, p<0.0001] and a significant interaction between chronic pretreatment and acute treatment [F(1,15) =  7.19, p = 0.0171]. Post hoc test showed that there was a significant increase (p<0.001) in the number of c-Fos positive neurons after naloxone-precipitated morphine withdrawal when compared with the control group receiving naloxone (Fig. 5G). Two-way ANOVA showed that acute treatment [F(1,15) = 12.06, p = 0.0034] had a significant effect on the number of TH positive neurons. Newman-Keuls post hoc test shows that there was a significant (p<0.05) lower number of TH positive neurons in morphine withdrawal rats treated with CP-154,526 versus the group treated with vehicle instead of CRF1 receptor antagonist (Fig. 5 H). To reveal if TH neurons are activated during naloxone-precipitated morphine withdrawal, c-Fos and TH immunostaining was co-localized and quantified in sections of the VTA. Two-way ANOVA revealed main effect of morphine pretreatment [F(1,15) = 29.05, p<0.0001] and acute treatment [F(1,15) = 31.06, p<0.0001]. Post hoc comparisons showed a significant (p< 0.001) increase in the number of TH-containing neurons expressing c-Fos after naloxone administration to morphine dependent rats (Fig. 5C, E, I). Pretreatment with CP-154,526 (20 mg/kg, i.p.) significantly (*p*<0.001) decreased the number of c-Fos positive cells and the number of TH containing neurons expressing c-Fos observed in morphine withdrawn rats treated with vehicle (Fig. 5C,D, E, F,I). In addition, the CRF1 receptor antagonist decreases the number of TH immunoreactivity neurons in the NAc (Fig. 5H).

## Discussion

Adaptive changes have been reported in a variety of signalling pathways and gene expression after acute and chronic morphine exposure, and during morphine withdrawal. However, it is during withdrawal syndrome when several of these adaptive changes become evident. In this regard, present data demonstrated that chronic morphine treatment did not change HPA axis activity, DA and NA turnover, TH phosphorylation and c-Fos expression. In contrast, these parameters were modified following naloxone-precipitated withdrawal.

Several studies have previously shown that increased CRF release contributes to the anxiety and aversive states produced by drug withdrawal [30], [31] and recruitment of the CRF system has been hypothesized to be involved in drug dependence [1]. Accordingly, antagonism of CRF neurotransmission attenuated the anxiety-like and aversive effects of drug withdrawal [14]. Consistent with these studies, we observed that CP-154,526 suppressed four behavior signs (rinorrhea, spontaneous jumping, salivation and diarrhoea). In agreement with present results, genetic disruption of CRF1 receptor pathways as well as pharmacological antagonism of the CRF1 receptor eliminates the negative affective states of opioid withdrawal [32], [33]. In addition, previous [33] and present results demonstrate that CP-154,526 attenuated the weight loss and others somatic signs of morphine withdrawal. In contrast, it has been showed that CRF1 receptor-deficient mice display increased somatic reactions to opiate withdrawal indicating a clear-cut dissociation between affective-like and somatic signs [34]. These opposite results could be explained by the different protocols used: CRF1 receptor-deficient mice versus rats pretreated with a selective CRF1 receptor antagonist.

According to previous reports, present findings demonstrated that naloxone administration to morphine dependent rats significantly elevated plasma ACTH and corticosterone concentrations. Enhanced responsiveness of the HPA axis after morphine withdrawal has been associated with activation of noradrenergic neurons in the NTS that project to the hypothalamic PVN, suggesting that one of the neuronal mechanisms that underlie morphine withdrawal-induced activation of the HPA axis may be dependent on activation of noradrenergic pathways innervating the PVN [35],[11],[12],[36]. Our results show that pretreatment with CP-154256 did not block the corticosterone release that is produced as a consequence of morphine withdrawal. However, ACTH concentrations were found to be decreased in animals pretreated with CP-154,526. The relationship between HPA activity and drug withdrawal-induced behaviour alterations and changes in the brain stress system has not been elucidated, and contradictory results have been shown. CRF1 receptor has been shown to play a major role mediating the effects of CRF on behavioural responses to lorazepam withdrawal but not some of the neuroendocrine effects. Thus according to present results, the CRF1 receptor antagonist R121919 attenuated the behavioural and autonomic signs of lorazepam withdrawal and ACTH secretion, whereas corticosterone levels were not altered [37]. Furthermore in clinical studies CRF1 receptor antagonists produced anxiolytic-like effects in depressed patients in the absence of effects on CRF-stimulated HPA axis activity [38]. A potential explanation to present findings is that, although CRF is thought to be a major secretagogue in stimulating ACTH secretion, arginine vasopressin (AVP), catecholamines, and other factors also play a role [39]. In addition, there is increasing evidence that AVP and CRF production and release from the parvocellular PVN neurons are under independent regulation. Thus, it is possible that AVP may play an important role in mediating the pituitary-adrenal response to drug withdrawal and stress [40].

DA signalling in the NAc is involved in the integration of sensory information and the initiation of the subsequent behavioural responses to diverse stimuli [41]. The perception and behavioural consequences of rewarding and aversive substrates mediating these opposing phenomena are unclear. According to the reward coding hypothesis [42]–[45], mesolimbic DA neurons are inhibited or unresponsive to aversive stimuli, and increased DA release only occurs with reward-related stimuli. However, electrophysiological studies clearly demonstrate that the activity of a subset of DA neurons in the VTA and substantia nigra can increase in response to aversive stimuli [46], [20], [47], [48]. Our data clearly demonstrate that naloxone-precipitated morphine withdrawal increases the activity of VTA TH-positive neurons (as reflected by c-Fos expression) concomitantly with an enhancement of DA turnover in the NAc, which receives projections from VTA, in parallel to increased TH phosphorylation (activation) at Ser40. According to these data, a previous study has demonstrated that dorsal striatum and NAc core are neural substrates, which are involved in the perception of aversive stimuli suggesting that DA response in these regions can be triggered by aversive states in the same fashion as rewarding stimuli [49].

While our data suggest that DA neurons in the VTA exhibit significant activation in response to an aversive stimulus that produce a proportional response in DA turnover and TH activation in the terminal region, others have reported that opioid withdrawal is associated with decreased activity of dopaminergic neurons and decreased DA output to the NAc [50], [51], [52]. Several factors may account for the contrasting results in the reward pathways, including different methods (microdialysis or electrophysiological studies versus HPLC or immunohistochemistry), withdrawal conditioned to the discontinuation of treatment or different doses of naloxone used by different groups.

On the other hand, direct noradrenergic inputs from the NTS to the NAc have been described [53]. The present findings demonstrated that naloxone administration to morphine dependent rats significantly elevated NA turnover in the NAc, which receives projections from NTS. Previous reports have shown that naloxone-precipitated morphine withdrawal increases the activity of NTS TH-positive neurons [12], [54]. Together, all these data implicated NA neurons in NTS in the aversiveness of acute opioid withdrawal. Support for this idea can be found in a number of studies that implicate the NTS-A2 noradrenergic cells group in that affective disorder associated with drug withdrawal [6], [54]–[56]. Changes in the state of phosphorylation of TH, the rate limiting enzyme in the synthesis of catecholamines, are critically involved in the regulation of catecholamines, synthesis and function. In particular, increases in the phosphorylation of Ser40 and Ser31 accelerate TH activity, thereby simulating production of neurotransmitter in catecholamines terminals [57]. Using phosphorylation state-specific antibodies directed toward Ser40 or Ser31, in the present study we have shown that naloxone-precipitated withdrawal greatly increased the level of TH phosphorylation at Ser40, without changes at Ser31, in NAc. Together, these data suggest that Ser40 phosphorylation of TH may be an important modulator of TH activity and might be directly involved with regulating NA and DA turnover in the NAc from morphine withdrawn rats. Short-term regulation of catecholamines biosynthesis occurs through phosphorylation of TH, which enhances enzymatic activity [58].

On the other hand, pretreatment with the selective CRF1 receptor antagonist CP-154,526 did not block the increased NA turnover in the NAc during morphine withdrawal suggesting that the activation of the CRF1 receptor subtype is not responsible for the elevation of NA neurotransmission innervating the NAc. Our results are consistent with findings from our laboratory demonstrating that CRF1 receptor is not implicated in the enhancement of NA turnover in the PVN, which receives projections from NTS, observed during morphine withdrawal [33]. In addition, a previous study using CRF1receptor-deficient mice demonstrated that CRF1 receptor is not responsible for the phosphorylation of TH observed in NTS after naloxone-precipitated withdrawal in wild-type mice [56].

On the other hand, we observed that the CRF1 receptor antagonist CP-154,526 decreased DA turnover in the NAc from morphine withdrawn rats in parallel with a decrease in the phosphorylation of TH at Ser40 in the NAc. According to these data, prior studies have shown that CRF1 receptor activation can increase DA cell firing and DA release in brain structures associated with reward, including the VTA and NAc [25], [58]. Since association of Ser40 phosphorylation with TH activity and catecholamine synthesis in vivo has been shown [59] present results indicate that CRF1 receptor is involved in TH phosphorylation observed in the NAc after naloxone-precipitated morphine withdrawal and suggest that TH activation is responsible for the enhancement of DA turnover.

Using dual immunolabeling for c-Fos (a marker of neuronal activity) and TH, present data show that TH positive neurons in the VTA coexpress c-Fos in morphine withdrawn rats. Present results show a decrease in the number of c-Fos positive cells and in TH positive neurons coexpressing c-Fos in the VTA from morphine withdrawn rats treated with CP-154,526. Altogether, these results support the idea that VTA dopaminergic neurons are activated in response to aversive stimulus and suggest that CRF1 receptors are involved in the activation of dopaminergic pathways which project to NAc.

Our data clearly demonstrated that naloxone-precipitated morphine withdrawal activated VTA dopaminegic neurons and also increased DA turnover in the NAc, which receives dense VTA input. Since DA responses in these anatomical structures can be triggered by aversive states in the same fashion as rewarding stimuli, our findings suggest the existence of some overlap in the neurochemistry of withdrawal and pleasure processing in the mesolimbic pathway. In addition, we showed that CRF1 receptor antagonist suppressed or attenuated some signs of morphine withdrawal, ACTH hypersecretion, DA turnover and TH phosphorylation in the NAc, in parallel with a decrease in the number of TH containing neurons expressing c-Fos in the VTA. Electrophysiological studies demonstrated that application of CRF increased VTA DA neuronal firing in a concentration dependent manner. However, the precise mechanism of CRF regulation of dopaminergic cells in the VTA is also unclear and has been reported to involve CRF1 receptor-mediated activation of protein kinase C (PKC) [28] and possibly protein kinase A (PKA) [60] signaling and/or CRF2 receptor facilitation of NMDA receptor function [61]. In addition, experiments using CRF agonists, CRF antagonists and CRF-receptor deficient mice all led to the same conclusion that CRF increased VTA DA neurons through CRF1 receptor activation [28]. According to these data, present results suggest that CRF could activate the mesolimbic DA system after morphine withdrawal via CRF1 receptor activation. This study identifies a link between DA and CRF, which together have been implicated in drug abuse and support a role for CRF1 receptors like a potential therapeutic target to treat this disorder.

## Materials and Methods

### Animals

Male Sprague-Dawley rats (220–240 g at the beginning of the experiments) were housed four-to-five per cage under a 12-h light/dark cycle (light: 8:00–20:00 h) in a room with controlled temperature (22±2°C), humidity (50±10%), food and water available *ad libitum* and prehandled for several days preceding the experiment to minimize stress. All surgical and experimental procedures were performed in accordance with the European Communities Council Directive of November 24, 1986 (86/609/EEC) and approved by the local committees for animal research (REGA ES300305440012), Murcia. The study was approved by the University of Murcia bioethics committee (RD 1201/2005) and Ministerio de Ciencia y Tecnología (SAF2010-17907), Spain.

### Drug treatment and experimental procedure

Rats were made dependent on morphine by subcutaneous implantation of two 75-mg morphine pellets (provided by the Ministerio de Sanidad, Madrid, Spain). This procedure has been shown to produce consistent plasma morphine concentration beginning a few hours after the implantation of the pellets and a full withdrawal syndrome after short-term treatment with opioid antagonists [62]. Dependence on morphine is achieved 24 h after implantation of pellets and remains constant for 15 days [63]. Control rats received placebo pellets containing the excipient without morphine. Six days after the implantation of morphine or placebo pellets, precipitated morphine withdrawal was induced by injection of naloxone (1 mg/kg, sc) or saline (controls) and the rats were observed for behavioural signs of withdrawal. The withdrawal symptoms (ptosis, teeth-chattering, tremor, piloerection, lacrimation, rinorrhea, spontaneous jumping, wet-dog shakes, salivation and diarrhoea) were observed after naloxone administration for a period of 30 min. In addition, body weight loss was determined as the difference between the weight determined immediately before saline or naloxone injection and a second determination made 60 min later. These signs are reliable markers of opiate withdrawal in morphine dependent rats. Rats weight gain was checked during the treatment to ensure that morphine was liberated correctly from the pellets because it is known that chronic morphine treatment induces a decrease in body weight gain due to lower caloric intake [64].

To determine the role of CRF1 receptor in the changes observed during morphine withdrawal, rats were treated with CP-154,526 [*N*-butyl-*N*-ethyl-2,5-dimethyl-7-(2,4,6-trimethyl-phenyl)pyrrolo(3,2-*e*)pyrimidin-4-amine] (20 or 30 mg/kg i.p., 30 min before naloxone or saline injection). The doses of CP-154,526, a selective CRF1 receptor antagonist, were selected based in a previous study from our laboratory [33]. These doses were dissolved in 10% Tween 80 and given in a volume of 1 ml/kg.

Sixty min after saline or naloxone injection, rats were decapitated. The brains were rapidly removed, fresh-frozen, and stored immediately at −80°C until use for Western Blot analysis and NA and DA turnover measurements. One set of each treatment group was randomly assigned for plasma ACTH and corticosterone determination.

### Radioimmunoassay

Plasma levels of corticosterone and ACTH were measured by commercially available kits for rats (^125^I-corticosterone and ^125^I-ACTH radioimmunoassay; MP Biomedicals, Orangeburg, NY). The sensitivity of the assay was 7.7 ng/ml for corticosterone and 5.7 pg/ml for ACTH.

### Estimation of NA, DA and their metabolites 3-methoxy-4-hydroxyphenylethylen glicol (MHPG) and 3,4-dihydroxyphenylacetic acid (DOPAC)

NA and its metabolite in the central nervous system MHPG, DA and its metabolite DOPAC were determined by high-performance liquid chromatography with electrochemical detection. Each tissue was weighed, placed in a dry cooled propylene vial and homogenized with a Polytron-type homogenizer in 1.5 mL perchloric acid (0.1 M). The homogenates were then centrifuged (8000 g, 4°C, 15 min), the supernatant layer was removed into a 1 mL syringe and filtered through a 0.45 mm filter (Millipore, Bedford, MA, USA) and centrifuged (6000 g, 4°C, 20 min) again through Ultra free MC 0.2 (Millipore). From each sample, 10 mL was injected into a 5 mm C18 reverse phase column (Waters, Milford, MA, USA) through a Rheodyne syringe-loading injector 200 µL loop. Electrochemical detection was accomplished with a glass carbon electrode set at a potential of +0.65 with respect to the Ag/AgCl reference electrode (Waters). The mobile phase consisted of a 95% (v/v) mixture of water and methanol with sodium acetate (50 mM), citric acid (20 mM), L-octyl-sodium sulphonate (3.75 mM), di-n-butylamine (1 mM) and EDTA (0.135 mM), adjusted to pH 4.3. The flow rate was 0.9 mL/min, and chromatographic data were analysed with Millenium 2010 Chromatography Manager Equipment (Millipore). NA, MHPG, DA and DOPAC were simultaneously detected by the described high-performance liquid chromatography method and were quantified by reference to calibration curves run at the beginning and at the end of each series of assays. Linear relationships were observed between the amount of standard injected and the peak height measured. The content of NA, DA and their metabolites in the NAc was expressed as ng/g protein. The NA or DA turnover was determined as the NA or DA ratio, which was calculated as follows: NA ratio = MHPG/NA, DA ratio = DOPAC/DA

### Western blot analysis

Western blot analysis was performed for THpSer31, THpSer40 and total TH determination. Samples were placed in homogenization buffer [phosphate buffered saline, 2% sodium dodecylsulfate (SDS), protease inhibitors (Roche, Germany) and phosphatase inhibitors (Calbiochem, Germany)] and homogenized for 50 s prior to centrifugation at 6000 g for 20 min at 4°C. Total protein concentrations were determined spectrophotometrically using the bicinchoninic acid method [65]. The optimum amount of protein to be loaded was determined in preliminary experiments by loading gels with increasing protein contents (25 to 100 µg) from samples of each experimental group. Equal amounts of protein (50 µg/lane) from each sample were loaded on a 10% SDS-polyacrylamide gel (SDS-PAGE), electrophoresed, and transferred onto a poly vinylidene difluoride (PVDF) membrane using a Mini Trans-Blot Electrophoresis Transfer Cell (Bio-Rad Lab., California, USA). Non-specific binding of antibodies was mitigated by incubating membranes in 1% bovine serum albumin (BSA) in tris buffer saline tween (TBST: 10 mM Tris-HCl, pH 7.6, 150 mM NaCl, 0.05% Tween 20). The blots were incubated overnight at 4°C, with the following primary antibodies: polyclonal anti-TH phosphoSer31 (1∶300 dilution; AB5423, Millipore, USA), or polyclonal anti-TH phosphoSer40 (1∶500 dilution; AB5935, Millipore, USA) in TBST with BSA. After extensive washings with TBST, the membranes were incubated for 1 h, at room temperature, with the peroxidase-labeled secondary antibody anti-rabbit sc-2004 (Santa Cruz, USA) at 1∶2500 dilution. After washing, immunoreactivity was detected with an enhanced chemiluminescent/chemifluorescent western blot detection system (ECL Plus, GE Healthcare, UK) and visualised by a Typhoon 9410 variable mode Imager (GE Healthcare). Antibodies were stripped from the blots by incubation with stripping buffer (glycine 25 mM and SDS 1%, pH2), for 1 h at 37°C. We used anti-total TH (1∶1000 dilution; AB152, Millipore, USA) as our loading control for all the experiments. The ratio of TH phosphoSer31/TH, TH phosphoSer40/TH was plotted and analysed.

Quantification of immunoreactivity corresponding to TH phosphoSer31 (60 kDa), TH phosphoSer40 (60 kDa) and TH (62 kDa) bands was carried out by densitometry (AlphaImager, Nucliber, Madrid). The optical density was normalized to the background values. Relative variations between bands of experimental samples and control samples were calculated in the same image.

### Immunohistochemistry. Double-labelling immunohistochemistry of c-Fos and TH-positive neurons in the VTA

Animals were perfused transcardiacally, and brains were processed for visualization of c-Fos and TH by using previously published techniques [66]. For c-Fos/TH double-label immunohistochemistry, brain stem tissue sections from each rat in each treatment group were processed as follows: c-Fos was revealed with 3,3′-diaminobenzidine (DAB) (Sigma Chemical, Madrid, Spain) intensified with nickel in the first position, and TH was revealed with DAB in the second position. Different group sections were incubated in primary polyclonal anti-total TH (1∶6000 dilution; AB152), polyclonal anti-(c-Fos) antibody (1∶10,000 dilution; non-cross-reactive with Fos-B, Fra-1 or Fra-2; sc-52; Santa Cruz) and a secondary anti-rabbit IgG (diluted 1∶500; Vector Laboratories) and c-Fos antibody-peroxidase complex was visualized by using a mixture of NiSO_4_.6H_2_O (33.2 mg/ml), DAB (0.033%), and 0,014% H_2_O_2_ in 0,175 M sodium acetate solution (pH 7.5). Tissue sections were transferred into Milli-Q water (Millipore Corporation) to stop the color reaction. Sections were mounted onto chrome-alum gelatin-coated slides, dehydrated, and coverslipped. The same immunohistochemistry procedures described above were followed for total TH. Positive nuclei for c-Fos immunoreactivity were detected using conventional light microscopy and counted at ×20 magnification. c-Fos-positive TH positive neurons were identified as cells with brown cytoplasmic deposits for TH-positive staining and blue/dark nuclear staining for c-Fos. A square field (129 µm) was superimposed on captured image for using as reference area. The number of single- and double-labeled c-Fos neurons observed bilaterally was counted in four to six sections from each animal in the VTA.

**Figure 6:**
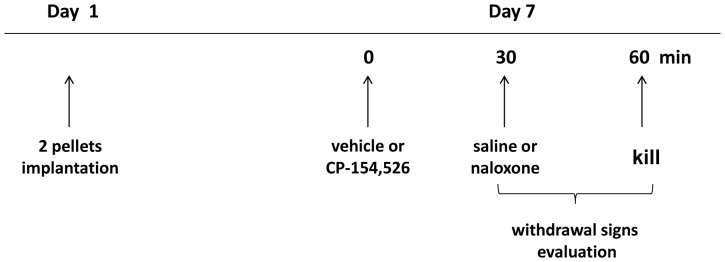
Experimental protocol. Six days after placebo or morphine pellets, rats were treated with CP or vehicle 30 min before saline or naloxone injection and 60 min after the injection, rats were decapitated or perfused transcardiacally for biochemical assays (as described under Material and Methods).

Figure 6 shows the experimental protocol used in the study.

### Drugs and Chemicals

Pellets of morphine base (Alcaliber Laboratories, Madrid, Spain) or lactose were prepared by the Department of Pharmacy and Pharmaceutic Technology (School of Pharmacy, Granada, Spain); sodium dodecylsulphate, polyacrylamide gel and PVDF membranes were obtained from Bio-Rad Laboratory (Teknovas, Bilbao, Spain); goat serum (Sigma Adrich, St. Louis, MO) and nickel sulfate (Sigma Adrich). Naloxone HCl was dissolved in sterile 0.9% NaCl (saline). CP-CP-154,526 (Sigma-Adrich) was dissolved in 10% Tween 80 (Sigma-Adrich). Drugs were prepared fresh every day.

### Statistical analysis

The results are expressed as the mean±S.E.M. using the GraphPad Prism statistical package. Body weight loss, and hormonal and biochemical parameters were analyzed by two-way analysis of variance (ANOVA) with pretreatment (placebo, morphine) and acute treatment (vehicle, CP-154,526) as independent variables. Newman-keuls post-hoc test was used for individual comparisons. Body weight gain in naive and morphine dependent rats were analysed by unpaired student's *t*-test. behaviours were quantified as the number of animals exhibiting the sign/total number of animals observed and data obtained were analyzed non-parametrically using the x^2^ test differences with a p value less than 0.05 were considered significant.
