# Correction: Role of Corticotropin-Releasing Factor (CRF) Receptor-1 on the Catecholaminergic Response to Morphine Withdrawal in the Nucleus Accumbens (NAc)

**DOI:** 10.1371/annotation/3708ead6-a61a-48b4-aca4-c45b3b7dd292

**Published:** 2013-05-17

**Authors:** Pilar Almela, Javier Navarro-Zaragoza, Juan-Antonio García-Carmona, Lucía Mora, Juana Hidalgo, María-Victoria Milanés, María-Luisa Laorden

There was an error in the Funding statement.

The correct statement is: This work was supported by Ministerio de Ciencia e Innovación (SAF/FEDER2010-17907), Red de Trastornos Adictivos (RTA; RD06/0001/1006) and Fundación Séneca (15405/PI/10), Región de Murcia. The funders had no role in study design, data collection and analysis, decision to publish, or preparation of the manuscript. 

